# Neural Network - Supported Prognostic Assessment of Apoptotic and Inflammatory Markers in Neovascular Glaucoma

**DOI:** 10.22336/rjo.2026.14

**Published:** 2026

**Authors:** Olga Volodymyrivna Guzun, Oleg Serhiyovich Zadorozhnyy, Camelia Margareta Bogdanici, Volodymyr Viktorovych Vychuzhanin, Liudmyla Mykolayivna Velichko, Andrii Rostyslavovich Korol, Valeriu Nicon Cușnir, Lilia Gheorghe Dumbrăveanu

**Affiliations:** 1 “The Filatov Institute of Eye Diseases and Tissue Therapy of the National Academy of Medical Sciences of Ukraine” State Institution, Odesa, Ukraine; 2 Department of Ophtalmology, “Grigore T. Popa” University of Medicine and Pharmacy, Iaşi, Romania; 3 “Odesa Polytechnic” National University, Odesa, Ukraine; 4 Department of Ophthalmology and Clinical Optometry, “Nicolae Testemițanu” State University of Medicine and Pharmacy, Chişinău, Republic of Moldova

**Keywords:** neovascular glaucoma, transscleral cyclophotocoagulation, CD95, CD54, AISI, SII, systemic inflammation, apoptosis, endothelial activation, prognosis, machine learning, AI = artificial intelligence, DR = diabetic retinopathy, HbA1c = glycated hemoglobin, PDR = proliferative diabetic retinopathy, NVG = neovascular glaucoma, TSCPC = transscleral cyclophotocoagulation, IOP = intraocular pressure, BCVA = best-corrected visual acuity, SII = systemic immune-inflammation index, SIRI = systemic inflammation response index, AISI = aggregate inflammation systemic index, ICAM-1 = intercellular adhesion molecule-1, CD54 = intercellular adhesion molecule-1, CD95 = Fas receptor (APO-1), PRP = panretinal photocoagulation

## Abstract

**Purpose:**

To assess the prognostic value of apoptotic (CD95), endothelial (CD54), and systemic inflammatory biomarkers (SII, SIRI, AISI) for predicting the effectiveness of modified selective diode transscleral cyclophotocoagulation (TSCPC) in patients with neovascular glaucoma using regression and neural network-based models.

**Materials and methods:**

This prospective cohort study included 258 eyes: 224 eyes with NVG (155 treatment successes and 69 failures) and 34 age-matched healthy controls. Baseline assessments comprised intraocular pressure (IOP), best-corrected visual acuity (BCVA, logMAR), and serum levels of CD95 (Fas), CD54 (ICAM-1), HbA1c, and systemic inflammatory indices (SII, SIRI, AISI). Treatment success was defined as IOP between 10 and 21 mmHg, stabilization or improvement of BCVA, and absence of additional glaucoma surgery. Between-group comparisons were performed using the Mann-Whitney U test and Dunn-Bonferroni post hoc analysis. Predictors of treatment failure were assessed using univariable and multivariable logistic regression. Discriminative performance was evaluated by receiver operating characteristic (ROC) analysis. A gradient-boosting machine-learning model with SHAP-based interpretability was applied to an independent validation set.

**Results:**

Patients with TSCPC failure exhibited significantly higher biomarker levels than those who had successful outcomes: CD95 (+41%), CD54 (+71%), and AISI (+164%) (all *p* < 0.001). In univariable analysis, CD95 (OR = 1.80; 95% CI: 1.55–2.15), AISI (OR = 1.60), and CD54 (OR = 1.50) showed the strongest associations with treatment failure. In the multivariable model, CD95 (OR = 1.65), CD54 (OR = 1.30), and AISI (OR = 1.40) remained independently significant, with robust explanatory power (Nagelkerke R^2^ = 0.60). The combined apoptosis-endothelial model (CD95 + CD54 + AISI) demonstrated superior discriminatory performance (AUC = 0.87; sensitivity 82%; specificity 76%). SHAP analysis confirmed that these three biomarkers accounted for approximately 75% of total predictive importance.

**Discussion:**

The findings of this study indicate that the effectiveness of modified selective TSCPC in neovascular glaucoma is determined not merely by baseline IOP, but predominantly by the patient’s integrated biological profile, particularly apoptotic (CD95), endothelial (CD54), and systemic inflammatory markers (SII, SIRI, AISI). The observed convergence of endothelial activation, systemic inflammation, and Fas-mediated apoptosis supports the concept of NVG as a multifactorial ischemic–inflammatory disorder and provides a mechanistic basis for treatment resistance. The combined biomarker model demonstrated high predictive performance (AUC = 0.87), outperforming individual parameters and underscoring the value of a multimarker approach. These findings support biomarker-guided risk stratification and reinforce the need for personalized therapeutic strategies, including early neuroprotective and anti-inflammatory interventions in patients at high risk of unfavorable outcomes.

**Conclusions:**

Apoptosis (CD95), endothelial activation (CD54), and systemic inflammation (AISI, SII, SIRI) constitute key biological determinants of resistance to TSCPC in NVG. An integrated CD95-CD54-AISI biomarker panel provides high prognostic accuracy and may support personalized risk stratification and treatment optimization in patients with neovascular glaucoma.

## Introduction

Neovascular glaucoma (NVG) is one of the most aggressive forms of secondary glaucoma that develops in the setting of profound retinal ischemia, endothelial dysfunction, and sustained angiogenic stimulation. Chronic retinal non-perfusion activates VEGF-dependent pathways alongside inflammatory and adhesion-mediated mechanisms, ultimately leading to iris rubeosis and secondary angle closure [1,2].

Despite the widespread use of anti-VEGF therapy and panretinal photocoagulation (PRP), clinical outcomes remain unfavorable in a substantial proportion of patients. In a retrospective cohort of patients with ischemic central retinal vein occlusion (CRVO), 21% of eyes developed anterior or posterior segment neovascularization within 20 months despite regular anti-VEGF injections [3]. Similarly, NVG was reported to occur in 13% of cases in an analysis of 646 patients [4]. These data indicate that current therapeutic strategies may delay, but do not fully prevent, pathological angiogenesis.

Importantly, ischemia and VEGF signaling stimulate not only angiogenesis but also the expression of endothelial adhesion molecules, particularly intercellular adhesion molecule-1 (ICAM-1, CD54). In experimental models of intravitreal VEGF administration, ICAM-1 expression increased sharply, accompanied by leukostasis and capillary non-perfusion; blockade of ICAM-1 reduced vascular leakage by up to 79% [5]. In ischemia-reperfusion models, ICAM-1 upregulation promoted leukocyte adhesion and structural retinal damage, whereas inhibition of adhesion molecules effectively prevented these changes [6]. Comparable mechanisms have been described in diabetic retinopathy (DR), where ICAM-1 activation reflects the microvascular and inflammatory components of the disease [7]. Thus, CD54 represents not only a biomarker but also an active mediator of the ischemic-inflammatory cascade and may serve as a predictor of NVG development.

Experimental ophthalmic studies further demonstrate a pivotal role of the Fas/FasL system in retinal ganglion cell (RGC) death. In models of acute ischemia and experimental glaucoma, activation of Fas/FasL signaling within the RGC layer triggers caspase-dependent apoptosis and rapid neuronal loss [8]. Similar apoptotic pathways have been identified in models of optic nerve axonal injury [9,10]. Contemporary studies demonstrate that Fas/FasL-mediated apoptosis is a key mechanism of RGC loss in response to chronic elevation of intraocular pressure (IOP) and associated neuroinflammation [8,11].

In clinical proliferative diabetic retinopathy (PDR), aqueous humor concentrations of Fas and FasL increase proportionally with neovascular activity and the severity of ischemic stress [12], supporting a close link between apoptosis, angiogenesis, and intraocular inflammation.

Taken together, apoptotic, endothelial-adhesive, and systemic inflammatory mechanisms create a complex biological substrate of NVG that appears to determine the effectiveness of transscleral diode cyclophotocoagulation (TSCPC) to a much greater extent than isolated mechanical ocular parameters. This multidomain concept leads to the central objective of the present study: to identify biomarkers that most accurately reflect this pathological cascade and to evaluate their prognostic value for treatment outcomes in a real-world NVG cohort.

### Aim of the study

To assess the prognostic value of apoptotic (CD95), endothelial (CD54), and systemic inflammatory biomarkers (SII, SIRI, AISI) for predicting the effectiveness of modified selective diode transscleral cyclophotocoagulation (TSCPC) in patients with neovascular glaucoma using regression and neural network-based models.

## Materials and methods

The overall study structure, measurement techniques, and analytical approaches are summarized in **Table 1**, providing a clear overview of cohort formation, biomarker assessment protocols, and the sequence of statistical and machine-learning analyses. The study design comprised three analytical levels: (1) comparative analysis of clinical and biological parameters across groups (control vs. NVG with treatment success vs. NVG with treatment failure); (2) univariate evaluation of potential predictors using logistic regression; and (3) multivariable modeling followed by independent validation using machine-learning techniques (gradient boosting with SHAP-based interpretation).

**Table 1:** Study structure, measurement methods, and analytical approaches

Component	Description / Methodology
Study design	Single-center prospective cohort study
Follow-up duration	24 months (assessments at V0, V12, V24)
Participants	258 eyes: 224 NVG eyes (155 treatment success; 69 TSCPC failure) and 34 healthy control eyes
NVG etiological subgroups	Proliferative diabetic retinopathy (NVG/PDR, n = 133) and retinal vein occlusion (NVG/RVO, n = 91)
Inclusion criteria	Clinically confirmed NVG (PDR or RVO); iris rubeosis Weiss grade ≥ 1; IOP > 25 mmHg; follow-up ≥ 12 months
Exclusion criteria	NVG of other etiologies; ocular surgery within ≤ 30 days before enrollment; severe systemic comorbidities precluding TSCPC
Ophthalmic examination	Intraocular pressure (Goldmann applanation tonometry); best-corrected visual acuity (BCVA, logMAR); slit-lamp biomicroscopy; gonioscopy; fundus examination
TSCPC technique	Modified selective diode transscleral cyclophotocoagulation; 20-24 applications; energy 1250-1500 mW; exposure time 2.0 s per application [13]; repeat procedures performed when clinically indicated
Biochemical markers (peripheral blood)	CD95 (Fas), CD54 (ICAM-1), systemic inflammatory indices (SII, SIRI, AISI), HbA1c
CD54 (ICAM-1) and CD95 (Fas) measurement	Flow cytometry; absolute counts of ICAM-1^+^ and Fas^+^ cells in peripheral blood (cells/µL)
Systemic inflammatory indices	SII = PLT × NEU / LYM; SIRI = NEU × MON / LYM; AISI = NEU × PLT × MON / LYM
Primary endpoint	TSCPC success: IOP 10-21 mmHg with sustained reduction from baseline; no additional glaucoma surgery; stabilization or improvement of BCVA; repeat TSCPC not classified as failure
Between-group comparisons	Mann-Whitney U test (success vs. failure); Kruskal-Wallis test with Dunn’s post hoc correction (PDR vs. RVO vs. control)
Correlation analysis	Spearman’s rank correlation (ρ) between biomarkers, IOP, and BCVA
Logistic regression	Univariate and multivariate models (final model: CD95 + CD54 + AISI)
Machine-learning modeling	Gradient Boosting Classifier
Model interpretation	SHAP (Shapley Additive Explanations)
ROC analysis	Individual models (CD95, CD54) and combined index (CD95 + CD54 + AISI)

Abbreviations: NVG = neovascular glaucoma, PDR = proliferative diabetic retinopathy, RVO = retinal vein occlusion, TSCPC = transscleral cyclophotocoagulation, IOP = intraocular pressure, BCVA = best-corrected visual acuity, SII = Systemic Inflammation Index, SIRI = Systemic Inflammation Response Index, AISI = Aggregate Inflammation Systemic Index, HbA1c = glycated hemoglobin, PLT = platelets, NEU = neutrophils, LYM = lymphocytes, MON = monocytes

### Study design and participants

This was a single-center prospective cohort study with a 24-month follow-up period, including assessments at baseline (V0), 12 months (V12), and 24 months (V24). A total of 258 eyes were enrolled: 224 eyes with neovascular glaucoma (NVG) and 34 eyes from healthy control subjects. Of NVG eyes, treatment success after TSCPC was achieved in 155 eyes, while 69 were treatment failures.

NVG eyes were stratified according to etiology into two subgroups: NVG secondary to proliferative diabetic retinopathy (NVG/PDR, n = 133) and NVG secondary to retinal vein occlusion (NVG/RVO, n = 91).

Inclusion criteria were: clinically confirmed NVG associated with PDR or RVO; iris rubeosis graded ≥ 1 according to the Weiss classification; intraocular pressure (IOP) > 25 mmHg; and a minimum follow-up duration of 12 months.

Exclusion criteria included NVG due to other etiologies, prior ocular surgery within 30 days before enrollment, and severe systemic comorbidities precluding TSCPC.

### Ophthalmic examination

All participants underwent comprehensive ophthalmic evaluation, including IOP measurement by Goldmann applanation tonometry, best-corrected visual acuity (BCVA) assessed in logMAR units, slit-lamp biomicroscopy, gonioscopy, and fundus examination.

### TSCPC procedure

All NVG eyes were treated using a modified selective diode transscleral cyclophotocoagulation technique. The procedure consisted of 20-24 laser applications with an energy setting of 1250-1500 mW and an exposure time of 2.0 seconds per application, in accordance with previously described parameters [13]. Repeat TSCPC was performed when clinically indicated and was not considered a treatment failure.

### Biomarker assessment

Peripheral venous blood samples were collected to assess apoptotic, endothelial, and systemic inflammatory markers. CD54 (ICAM-1) and CD95 (Fas) levels were quantified by flow cytometry and expressed as absolute counts of ICAM-1^+^ and Fas^+^ cells per microliter of peripheral blood (cells/µL).

Systemic inflammatory indices were calculated using standard formulas: SII = PLT × NEU / LYM; SIRI = NEU × MON / LYM; AISI = NEU × PLT × MON / LYM.

Glycemic control was assessed by measuring glycated hemoglobin (HbA1c).

### Outcome measures

The primary endpoint was TSCPC success, defined as an IOP between 10 and 21 mmHg with a sustained reduction from baseline, absence of additional glaucoma surgery, and stabilization or improvement of BCVA. Repeat TSCPC procedures were not classified as treatment failure.

### Statistical analysis

Statistical analysis was conducted at multiple levels. Between-group comparisons were performed using the Mann-Whitney U test for two-group analyses and the Kruskal-Wallis test with Dunn’s post hoc correction for multiple comparisons across the three-group structure (PDR, RVO, control). Correlations between biomarkers, IOP, and BCVA were assessed using Spearman’s rank correlation coefficient (ρ).

Univariate and multivariate logistic regression models were applied to identify predictors of TSCPC outcome. The final multivariable model included CD95, CD54, and AISI as representative markers of apoptosis, endothelial activation, and systemic inflammation, respectively.

For independent validation, machine-learning analysis was performed using a Gradient Boosting Classifier. Model interpretability was achieved through SHAP (Shapley Additive Explanations), enabling assessment of feature importance and directionality. Receiver operating characteristic (ROC) analysis was conducted for individual biomarkers (CD95, CD54) and for the combined apoptotic–endothelial index (CD95 + CD54 + AISI).

### Ethical considerations

The study was approved by the Bioethics Committee of the Filatov Institute of Eye Diseases and Tissue Therapy (Protocol No. 4, 2024). Written informed consent was obtained from all participants in accordance with the Declaration of Helsinki.

## Results

### Baseline characteristics and between-group differences

The study included 258 patients (258 eyes): 224 eyes with NVG, comprising 133 eyes with NVG secondary to proliferative diabetic retinopathy (NVG/PDR) and 91 eyes with NVG secondary to retinal vein occlusion (NVG/RVO), all of whom underwent modified selective diode TSCPC, as well as 34 eyes from an age-matched healthy control group.

Within the NVG cohort, 155 eyes (69.2%) achieved sustained IOP control with stabilization or improvement in BCVA and were classified as the treatment success group, whereas 69 eyes (30.8%) met the predefined criteria for treatment failure.

The control group exhibited significantly lower levels of systemic inflammatory indices (SII, SIRI, AISI), markers of endothelial activation (CD54), apoptotic activity (CD95), and IOP compared with both etiological NVG subgroups (all *p* < 0.0001, Kruskal-Wallis test).

Patients with TSCPC failure demonstrated significantly higher baseline levels of the apoptotic marker CD95, the endothelial activation marker CD54, and all integral systemic inflammatory indices (SII, SIRI, AISI) compared with patients who achieved treatment success (all *p* < 0.0001). Similarly, baseline IOP and HbA1c levels were significantly higher in the failure group. The distribution of baseline clinical and biological parameters in the two NVG outcome groups is summarized in **Table 2**.

**Table 2:** Baseline characteristics and between-group differences according to TSCPC outcome

Parameter	TSCPC success (n = 155)	TSCPC failure (n = 69)	p value
**Median (Q1–Q3)**	
SII (ratio)	395.8 (369.6-430.5)	639.2 (525.9-672.0)	<0.0001
SIRI (ratio)	0.541 (0.498-0.636)	1.128 (0.933-1.137)	<0.0001
AISI (ratio)	106.7 (93.7-124.0)	282.2 (236.8-315.5)	<0.0001
HbA1c, %	6.4 (4.4-7.2)	8.8 (4.4-9.5)	<0.0001
IOP V0, mmHg	33 (32-36)	40 (38-42)	<0.0001
IOP V24, mmHg	19 (17-21)	24 (20-28)	<0.001
CD54 (ICAM-1), cells/µL	353 (334-378)	605 (566-644)	<0.0001
CD95 (Fas), cells/µL	610 (553-639)	862 (798-921)	<0.0001
BCVA V0 (logMAR)	1.40 (1.70-1.10)	1.70 (2.00-1.70)	<0.001
BCVA V24 (logMAR)	1.15 (1.40-0.77)	2.00 (2.30-2.00)	<0.001

Note: Data are presented as median (Q1-Q3). *p-values* indicate statistical significance of between-group differences assessed using the Mann-Whitney U test. Abbreviations: SII = Systemic Inflammation Index, SIRI = Systemic Inflammation Response Index, AISI = Aggregate Inflammation Systemic Index, HbA1c = glycated hemoglobin, IOP = intraocular pressure, CD54 (ICAM-1), cells/µL = absolute count of ICAM-1^+^ cells in peripheral blood, CD95 (Fas), cells/µL = absolute count of Fas^+^ cells in peripheral blood, BCVA = best-corrected visual acuity (logMAR)

### Functional outcomes (BCVA, logMAR)

In the TSCPC success group, a statistically significant improvement or stabilization of visual function was observed. The median baseline BCVA was 1.40 logMAR and improved to 1.15 logMAR at 24 months. The interquartile ranges (1.70-1.10 at V0 and 1.40-0.77 at V24) indicated that a subset of eyes achieved clinically meaningful visual improvement.

At baseline, 19% of eyes in this group had no light perception; during follow-up, one additional eye progressed to complete vision loss by 24 months.

In contrast, the TSCPC failure group presented with substantially worse baseline visual acuity (median 1.70 logMAR; range 2.00-1.70), which continued to deteriorate over time, reaching a median of 2.00 logMAR at 24 months (range 2.30-2.00). In this group, no light perception was documented in 43% of eyes at baseline, increasing to 55% after 24 months.

### Between-group differences in apoptotic, endothelial, and systemic inflammatory biomarkers

Comparative analysis of outcome groups demonstrated that successful TSCPC was associated with stabilization or partial improvement of visual function. In contrast, a pronounced apoptotic-inflammatory profile (CD95/CD54/AISI) was linked to persistently poor visual acuity, even after repeated laser interventions.

Relative increases in median biomarker levels in the TSCPC failure group compared with the success group were as follows:

- CD95: +41%, indicating markedly increased apoptotic activity;
- CD54: +71%, reflecting pronounced endothelial activation and dysfunction;
- AISI: +164%, corresponding to an almost threefold increase in systemic inflammatory burden.

Post hoc Dunn-Bonferroni analysis revealed that elevated CD95 and CD54 levels were characteristic of the ischemic-neovascular NVG phenotype and were independent of NVG etiology (PDR vs. RVO). In contrast, the control group consistently exhibited low levels of these biomarkers, supporting their role as systemic pathogenetic markers rather than purely local ocular phenomena.

### Univariate analysis of predictors of TSCPC failure

Univariate logistic regression analysis demonstrated that all investigated biomarkers and clinical parameters were significantly associated with the risk of TSCPC failure (*p* < 0.001), underscoring the multicomponent nature of NVG pathogenesis and indicating that unfavorable outcomes arise at the intersection of several pathological domains.

The strongest associations with treatment failure were observed for biomarkers of apoptosis, endothelial activation, and systemic inflammation. CD95 exhibited the highest prognostic strength (OR = 1.80; 95% CI: 1.55-2.15), followed by AISI (OR = 1.60; 95% CI: 1.38-1.89), CD54 (OR = 1.50; 95% CI: 1.29-1.77), and SII (OR = 1.45; 95% CI: 1.31-1.63). These findings indicated that excessive apoptotic signaling, endothelial dysfunction, and systemic inflammatory load were key determinants of resistance to TSCPC.

Among conventional clinical parameters, baseline intraocular pressure remained a significant modifier of failure risk (OR = 1.25; 95% CI: 1.11-1.41), as did HbA1c levels (OR = 1.40; 95% CI: 1.18-1.62). Both parameters suggested that uncontrolled hyperglycemia and elevated IOP imposed additional stress on ischemia-compromised anterior segment tissues and exacerbated neurovascular dysfunction.

Overall, univariate analysis indicated that unfavorable TSCPC outcomes were driven not only by local ocular alterations but also by integrated systemic mechanisms involving apoptosis, endothelial activation, immune-inflammatory cascades, and metabolic dysregulation. This composite risk profile highlighted the need for a personalized, biomarker-guided approach to managing patients with neovascular glaucoma.

### Multivariable logistic regression model

The multivariable logistic regression model included three key pathobiological markers -CD95, CD54, and AISI - selected based on maximal prognostic informativeness and minimal multicollinearity.

The final adjusted model demonstrated that all three variables remained independently associated with TSCPC failure:

- CD95: adjusted OR = 1.65 (95% CI: 1.40-1.98), *p* < 0.001
- CD54: OR = 1.30 (95% CI: 1.09-1.56), *p* = 0.004
- AISI: OR = 1.40 (95% CI: 1.18-1.68), *p* = 0.001

The model exhibited strong explanatory power (Nagelkerke R^2^ = 0.60) and good calibration (Hosmer-Lemeshow test, *p* > 0.20). These results indicated that the combined burden of apoptotic activation, endothelial dysfunction, and systemic inflammation defined the most unfavorable prognostic profile for TSCPC outcomes.

### Gradient boosting model and SHAP analysis

The Gradient Boosting machine-learning model corroborated the dominant role of the apoptotic-endothelial axis in predicting TSCPC failure.

According to SHAP-based feature attribution, the relative contributions to model predictions were as follows: CD95 - 33%, CD54 - 24%, AISI - 18%, and SII - 12%.

Overall model performance was high, with an area under the ROC curve (AUC) of 0.87, sensitivity of 82%, and specificity of 76%.

Thus, machine-learning validation consistently confirmed the leading prognostic importance of apoptosis (CD95) and endothelial activation (CD54), whereas intraocular pressure and HbA1c acted as secondary modifiers within an already established pathogenic framework.

### ROC analysis of individual biomarkers and the combined model

ROC analysis revealed heterogeneous discriminatory performance among individual biomarkers (**Table 3**).

**Table 3:** ROC analysis of apoptotic, endothelial, and combined predictive models

Marker / Model	AUC	Optimal cutoff (Youden J)	Sensitivity (%)	Specificity (%)
CD95 (Fas), cells/µL	0.84	780	81	76
CD54 (ICAM-1), cells/µL	0.79	520	75	70
AISI (ratio)	0.81	180	78	72
Combined model (CD95 + CD54 + AISI)	0.87	Youden J = 0.58	82	76

Abbreviations: CD95 (Fas), cells/µL = absolute count of Fas^+^ cells in peripheral blood, CD54 (ICAM-1), cells/µL = absolute count of ICAM-1^+^ cells in peripheral blood, AISI = Aggregate Inflammation Systemic Index, AUC = area under the receiver operating characteristic curve

**Fig. 1:**
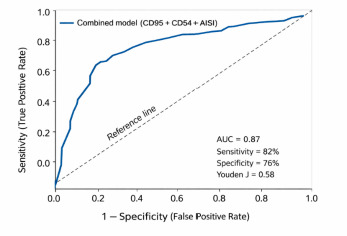
Receiver operating characteristic (ROC) curve of the combined apoptotic-endothelial model (CD95 + CD54 + AISI) for predicting failure of transscleral cyclophotocoagulation. The model demonstrated good discriminatory performance with an area under the curve (AUC) of 0.87, sensitivity of 82%, specificity of 76%, and an optimal cutoff determined by the Youden index (J = 0.58)

Among individual biomarkers, CD95 demonstrated the highest discriminatory ability (AUC = 0.84), followed by CD54 (AUC = 0.79). However, the highest overall predictive accuracy was achieved by the combined apoptotic-endothelial model (CD95 + CD54 + AISI), which yielded an AUC of 0.87 with balanced sensitivity (82%) and specificity (76%). The corresponding ROC curve is presented in **Fig. 1**.

## Discussion

In this study, we analyzed systemic and local biological determinants of response to modified selective TSCPC in patients with NVG. The overall treatment success rate was 69.2%, which is consistent with previously reported clinical series of TSCPC in NVG, where success rates typically range from 54.5% to 70% [14-16]. Importantly, intergroup differences observed in our cohort clearly indicate that the outcome of modified TSCPC is driven predominantly by the patient’s biological profile - specifically apoptotic, endothelial, and inflammatory markers - rather than by isolated mechanical ocular parameters such as baseline IOP alone [4,17,18].

### NVG as a multisystem inflammatory-endothelial and apoptotic syndrome

Comparison with the control group revealed pronounced differences in CD54, CD95, and systemic inflammatory indices (SII, SIRI, AISI), with levels being 2.5-4-fold higher in NVG patients. These findings support the contemporary concept that NVG represents not merely a consequence of local retinal ischemia but rather a manifestation of systemic immune-vascular dysregulation [17,19].

Elevated SII and AISI values reflect activation of the platelet-leukocyte inflammatory cascade, a typical response to systemic inflammatory stress that promotes microvascular occlusion and progression of retinal ischemia. Concurrently, increased CD54 (ICAM-1) levels indicate marked endothelial activation, facilitating leukostasis, microcirculatory impairment, and tissue hypoxia. Funatsu et al. demonstrated that in diabetic ischemic retinal disease, elevated ICAM-1 levels in intraocular fluids are associated with greater retinal nonperfusion, angiogenic activity, and inflammatory burden [20]. Similar observations were reported by Yao and colleagues, who identified ICAM-1 as a key mediator of leukostasis and microvascular damage in ischemic ophthalmopathies [21].

Taken together, the coexistence of elevated SII/AISI and CD54 in our cohort likely reflects early establishment of a complex vascular-inflammatory phenotype, underscoring the systemic nature of ischemic ophthalmopathies and potentially explaining reduced efficacy of local interventions in NVG.

### CD95 as a leading marker of early neurodegeneration and a determinant of TSCPC response

CD95 (Fas) is widely recognized as a critical mediator of early ischemia-induced neurodegeneration, and our findings confirm its strong prognostic relevance. In our cohort, CD95 levels in the TSCPC failure group were approximately 40-45% higher than in the success group and more than threefold higher than in controls, indicating pronounced activation of Fas-dependent apoptosis.

These observations are consistent with experimental models of ischemia and glaucoma, in which Fas/FasL signaling is a key trigger of caspase-dependent retinal ganglion cell (RGC) apoptosis in response to ischemia, oxidative stress, and axonal injury [22,23]. Mogilevskyi et al. demonstrated that elevated intraocular levels of apoptotic markers (TNF-α, FasL, sFas/APO-1) are associated with an increased risk of RGC loss progression, emphasizing the pivotal role of apoptosis in glaucomatous damage independently of IOP [24].

Our clinical findings align with this concept: elevated CD95 was associated with worse BCVA outcomes, reflecting accelerated RGC neurodegeneration. In the context of NVG, this mechanism is particularly critical, as NVG combines acute or subacute retinal ischemia, pronounced inflammatory stress, and rapid IOP elevation-factors that synergistically amplify Fas-mediated apoptotic cascades in RGCs [23].

From a clinical perspective, increased Fas activation in patients with preserved BCVA should be interpreted as a marker of “subclinical” neurodegenerative activity, signaling the need for early initiation of neuroprotective therapy and stricter IOP control strategies. This approach aligns with current neuroprotection paradigms in glaucoma, which emphasize early, targeted intervention in apoptotic, oxidative, and mitochondrial pathways before irreversible neuronal loss [25,26].

### The role of endothelial activation and systemic inflammation in resistance to TSCPC

CD54 (ICAM-1) and integral inflammatory indices (SII, SIRI, AISI) demonstrated robust intergroup differences and consistent associations with treatment failure. These findings are concordant with evidence that leukocyte adhesion, leukostasis, and microcirculatory impairment critically exacerbate anterior segment ischemia and promote the formation of dense neovascular membranes [20].

Notably, even at comparable IOP levels, patients with elevated CD54 or AISI exhibited significantly worse outcomes. This observation shifts the traditional paradigm from a purely mechanical interpretation (“high IOP → low success”) toward a biological model of resistance, in which endothelial dysfunction, systemic inflammation, and microcirculatory failure determine the effectiveness of TSCPC [27].

A similar inflammatory phenotype has been described in patients with diabetic macular edema after pars plana vitrectomy, where aqueous humor levels of IL-6, IL-8, IP-10, and MCP-1 were markedly elevated despite reduced VEGF concentrations, indicating a shift toward VEGF-independent inflammatory cascades [28]. These parallels further support the concept that inflammatory and endothelial mechanisms critically modulate treatment response in ischemic retinal diseases.

### Advantages of the combined apoptotic-endothelial-inflammatory model

The combined model incorporating CD95, CD54, and AISI demonstrated the highest discriminative performance (AUC = 0.87), surpassing that of any single biomarker. Machine-learning analysis confirmed that these three markers accounted for the majority of predictive contribution, whereas IOP and HbA1c acted as secondary modifiers.

This finding is consistent with emerging trends emphasizing integration of systemic and ophthalmic parameters to achieve more accurate prognostic stratification than reliance on a single pathological domain [29,30]. Our data underscore the multidimensional nature of resistance to TSCPC. In this apoptosis, endothelial activation, and systemic inflammation form a unified pathogenic continuum that governs treatment response, as suggested by prior investigations [31].

Experimental evidence further illustrates the time-dependent vulnerability of RGCs to ischemic injury: retinal ischemia lasting 45 minutes induces degeneration of approximately 50% of neurons within two weeks, whereas 120 minutes of ischemia results in loss of up to 99% within three months [32]. These data highlight the narrow therapeutic window for effective intervention in ischemic neurodegeneration.

### Clinical implications: toward personalized management of NVG

Our findings delineate several key directions for personalized NVG management. First, TSCPC remains a justified and effective treatment option in NVG [33], particularly when combined with PRP or anti-VEGF therapy [34]. Second, biomarker-guided risk stratification enables identification of patients with elevated CD95, CD54, and AISI who are at high risk of TSCPC resistance; in such cases, early incorporation of anti-inflammatory and neurotrophic therapies may be warranted.

Third, early neuroprotection emerges as a rational strategy: the combination of elevated CD95 and preserved BCVA suggests occult neurodegenerative activity and may warrant neuroprotective interventions [26,35]. Importantly, multidisciplinary optimization of systemic factors-glycemic control, lipid profile management, and reduction of chronic subclinical inflammation-may attenuate neovascular stimuli and slow disease progression.

Finally, for patients at high risk of treatment failure, combined therapeutic strategies integrating TSCPC with PRP, anti-VEGF therapy, systemic factor correction, and anti-inflammatory agents may provide a more comprehensive approach to the vascular-inflammatory and apoptotic components of NVG.

Several limitations of this study warrant consideration. Treatment outcomes were assessed exclusively for modified selective TSCPC and were not directly compared with alternative surgical approaches, such as Ahmed glaucoma valve implantation. In addition, data collection was coordinated primarily through a single center. Nevertheless, the study was conducted within a collaborative research framework involving investigators from Odessa, Chișinău, and Iași, thereby enhancing methodological consistency and external clinical relevance. Notable strengths include a large and clinically homogeneous cohort, an age-matched control group, comprehensive evaluation of three key pathogenic domains (apoptosis, endothelial activation, and systemic inflammation), and the integration of machine-learning-based analytical methods. Future studies should extend this collaboration into fully multicenter prospective investigations to validate the proposed biomarker-driven prognostic model across diverse patient populations.

## Conclusion

1. Neovascular glaucoma is associated with pronounced systemic apoptotic, inflammatory, and endothelial imbalance.
  Levels of CD95 were increased 3.4-fold, CD54 2.8-fold, and systemic inflammatory indices (SII, SIRI, AISI) 2.5-4-fold compared with controls (*p* < 0.0001), confirming the multifactorial and systemic nature of the ischemic-neovascular process.
2. Failure of transscleral cyclophotocoagulation is linked to a high-risk apoptotic-inflammatory phenotype.
  Patients with TSCPC failure demonstrated significantly elevated levels of CD95 (+41%; 862 vs. 610 cells/µL), CD54 (+71%; 605 vs. 353 cells/µL), and AISI (nearly twofold increase), reflecting marked activation of apoptotic pathways and endothelial dysfunction (*p* < 0.001). This biological profile differed substantially from that observed in patients with successful IOP control after TSCPC, supporting the pivotal role of apoptotic-inflammatory mechanisms in treatment resistance.
3. Multivariable analysis confirmed the independent contribution of three key pathogenic domains.
  Apoptosis (CD95), endothelial activation (CD54), and systemic inflammation (AISI) remained significant predictors in the final regression model (CD95: OR = 1.65; CD54: OR = 1.30; AISI: OR = 1.40), indicating an autonomous effect of each mechanism on the likelihood of TSCPC resistance. The model demonstrated strong explanatory power (Nagelkerke R^2^ = 0.60) and good calibration (*p* > 0.2), supporting its prognostic robustness in clinical practice.
4. Machine-learning-based integration further enhanced predictive accuracy. In the Gradient Boosting model, CD95, CD54, and AISI constituted the dominant block of prognostic determinants, accounting for up to 75% of the model’s total informativeness. The combined model achieved high discriminatory performance (AUC = 0.87, sensitivity = 82%, specificity = 76%), significantly outperforming individual biomarkers and conventional clinical parameters.
